# The Mediating Effect of Gaming Motivation Between Psychiatric Symptoms and Problematic Online Gaming: An Online Survey

**DOI:** 10.2196/jmir.3515

**Published:** 2015-04-07

**Authors:** Orsolya Király, Róbert Urbán, Mark D Griffiths, Csilla Ágoston, Katalin Nagygyörgy, Gyöngyi Kökönyei, Zsolt Demetrovics

**Affiliations:** ^1^Institute of PsychologyEötvös Loránd UniversityBudapestHungary; ^2^Doctoral School of PsychologyEötvös Loránd UniversityBudapestHungary; ^3^International Gaming Research Unit, Psychology DivisionSocial SciencesNottingham Trent UniversityNottinghamUnited Kingdom

**Keywords:** video games, Internet, motivation, behavior, addictive, psychopathology, coping behavior

## Abstract

**Background:**

The rapid expansion of online video gaming as a leisure time activity has led to the appearance of problematic online gaming (POG). According to the literature, POG is associated with different psychiatric symptoms (eg, depression, anxiety) and with specific gaming motives (ie, escape, achievement). Based on studies of alcohol use that suggest a mediator role of drinking motives between distal influences (eg, trauma symptoms) and drinking problems, this study examined the assumption that there is an indirect link between psychiatric distress and POG via the mediation of gaming motives. Furthermore, it was also assumed that there was a moderator effect of gender and game type preference based on the important role gender plays in POG and the structural differences between different game types.

**Objective:**

This study had two aims. The first aim was to test the mediating role of online gaming motives between psychiatric symptoms and problematic use of online games. The second aim was to test the moderator effect of gender and game type preference in this mediation model.

**Methods:**

An online survey was conducted on a sample of online gamers (N=3186; age: mean 21.1, SD 5.9 years; male: 2859/3186, 89.74%). The Brief Symptom Inventory (BSI), the Motives for Online Gaming Questionnaire (MOGQ), and the Problematic Online Gaming Questionnaire (POGQ) were administered to assess general psychiatric distress, online gaming motives, and problematic online game use, respectively. Structural regression analyses within structural equation modeling were used to test the proposed mediation models and multigroup analyses were used to test gender and game type differences to determine possible moderating effects.

**Results:**

The mediation models fitted the data adequately. The Global Severity Index (GSI) of the BSI indicated that the level of psychiatric distress had a significant positive direct effect (standardized effect=.35, *P*<.001) and a significant indirect (mediating) effect on POG (standardized effect=.194, *P*<.001) via 2 gaming motives: escape (standardized effect=.139, *P*<.001) and competition (standardized effect=.046, *P*<.001). The comparison of the 2 main gamer types showed no significant differences in the model. However, when comparing male and female players it was found that women had (1) slightly higher escape scores (on a 5-point Likert scale: mean 2.28, SD 1.14) than men (mean 1.87, SD 0.97) and (2) a stronger association between the escape motive and problematic online gaming (standardized effect size=.64, *P*<.001) than men (standardized effect size=.20, *P*=.001).

**Conclusions:**

The results suggest that psychiatric distress is both directly and indirectly (via escape and competition motives) negatively associated with POG. Therefore, the exploration of psychiatric symptoms and gaming motives of POG can be helpful in the preparation of prevention and treatment programs.

## Introduction

The large-scale expansion of the video game industry and online video gaming as a leisure time activity [1] has led to the appearance of problematic online gaming (POG) and online gaming addiction [2,3]. Although only a minority of players appear to display addiction-like symptoms and negative consequences on other important activities (ie, work, education) and relationships [4], it is still essential to explore the contributing factors in the development of this behavior. The importance of the phenomenon is also demonstrated by research findings that claim that online gaming is generally the most frequent problem associated with Internet use [5] and by the fact that POG was recently introduced into Section 3 of the fifth edition of the *Diagnostic and Statistical Manual of Mental Disorders* (*DSM-5*) [6] under the name of “Internet Gaming Disorder.” Hopefully its inclusion will encourage further research on the topic and help unify the field [7].

The attraction of online games appears to lie in their potential to satisfy different psychological needs that can be conceptualized as motives for gaming. In one earlier study, 7 motivational dimensions for online gaming were identified [8]. *Social motivation* concerns the need of gaming together with others and making friends. *Escape* refers to gaming in order to avoid real life problems and difficulties. *Competition* concerns the defeating of others, whereas *skill development* is about improving the player’s own coordination, concentration, and other skills. *Coping* involves stress, tension, or aggression reduction through gaming as well as getting into a better mood. *Fantasy* refers to trying out new identities and/or activities in virtual game words that are not possible in the gamers’ everyday lives. Finally, *recreation* concerns gaming for the playing of the game for fun. In fact, a recent study demonstrated that self-reported gaming motives were related to actual in-game behaviors and could also predict future in-game behaviors [9].

Studies into alcohol use have shed light on the importance of motives in both drinking and problem drinking (eg, [10]). Similarly, an association between different gaming motives and POG has been reported in the literature. Yee [11] found that escapism and advancement motives were the best predictors of problematic use of massively multiplayer online role-playing games (MMORPGs). Nagygyörgy and colleagues [12] yielded similar results (ie, escapism and achievement showed strong association with problematic MMORPG play). However, Zanetta Dauriat and colleagues [13] found that addictive MMORPG use patterns were best predicted by achievement, escapism, and socializing motives. Kwon and colleagues [14] suggested that adolescents become addicted to online games in an attempt to escape from self and reality based on Baumeister’s [15] escape theory.

It has also been reported that psychiatric symptoms can be related to POG. For instance, depression, anxiety, and social anxiety are positively related to problematic gaming [16-20]. An association has also been found between various domains of psychopathology and problem video gaming using the Symptom Checklist 90 (SCL-90) [21].

To date, psychiatric symptoms have been viewed as being directly related to POG. This approach is understandable because—as in other addictions—it is highly probable that psychiatric symptoms accompany the problem behavior. However, an indirect link might also exist between the 2 entities via the mediation of specific motivations. Such findings have been reported in the alcohol and gambling literature. Drinking motives have been shown to be the most proximate factors that precede alcohol use, the gateway via which more distal influences (eg, alcohol expectancies, anxiety sensitivity, trauma symptoms, and personality) are mediated [22-26]. For instance, the coping motive mediates between tension-reduction expectancies and drinking problems [22]. Moreover, in the case of gambling, the motivation to escape or dissociate has been found to mediate between aversive physiological and emotional states and gambling severity [27,28].

Gender differences may also play an important role in problematic gaming. Research has consistently shown that males are more likely to play online games [4,29,30] and spend significantly more time gaming than female players [31,32]. The literature also suggests that male players are at higher risk of problematic gaming than female players [4,30,32,33]. Furthermore, males and females also differ regarding their game type preference and motives to play. For instance, male gamers tend to prefer action, sports, and shooter games, whereas female gamers prefer puzzle, adventure, and quiz games [34,35]. It has also been found that male gamers score higher on motives related to achievement and competition, whereas female gamers score higher on social motives and escapism [8,11]. These findings are in-line with gender differences in competitiveness and social-emotional inclination. Males tend to be more competitive in various contexts, such as between-group interactions [36] and negotiations [37]. In contrast, females have been considered to be more interpersonally oriented [38,39]. In addition, females are more prone to experience negative emotions and to internalize problems reflected in higher prevalence of depression [40] and anxiety disorders [41], and this is in-line with their higher escapism scores. It has also been argued that clinical correlates of gaming may differ by gender [17].

Given that different game types (eg, role-playing games, first-person shooters, and real-time strategy games) vary in their basic structural characteristics and gameplay [42,43], it is reasonable to assume that psychiatric symptoms and motives related to different game types might also vary. In-line with this assumption, Stetina and colleagues [44] found that MMORPG players showed higher tendency toward depressive symptoms and escapism than gamers playing online shooters or real-time strategy games.

Consequently, the present study had 2 aims. The first aim was to test the mediating role of online gaming motives between psychiatric symptoms and problematic use of online games. Based on the literature, it was assumed that escape, coping, and competition motives would act as mediators in the model. The second aim was to test the moderator effect of gender and game type preference in this mediation model. Based on gender differences reported in the online gaming literature, differences in the paths related to competition, social, and escape motives have been expected between male and female players. On the other hand, no assumptions have been made regarding game type-related differences in the lack of findings in this domain.

## Methods

The study was approved by the Institutional Review Board of the Eötvös Loránd University, Budapest, Hungary.

### Recruitment

All the most frequently visited Hungarian online gaming websites having more than 100 registered users were identified (N=18). The majority of the sites (n=11) were game-specific: (massively) multiplayer online role playing games ([M]MORPGs) (eg, Lineage II, World of Warcraft, Guild Wars, Diablo II, Lord of the Rings; Thrillion kincsei [The Treasures of Thrillion])*;* (massively) multiplayer online real-time strategy ([M]MORTS) games (eg, Age of Empires 3, Travian, Klánháború [TribalWars], and Red Alert 3)*;* multiplayer online first-person shooter (MOFPS) games (eg, Call of Duty), 1 site was genre-specific (ie, role-playing games), and 6 sites were general video gaming sites. The search was carried out by 4 experts using online search engines (ie, Google) and additionally obtaining information from gamers from their circle of acquaintances. Subsequently, the 18 websites were invited to advertise for participants in the present study. All administrators agreed to cooperate and advertised the call for participation on their websites or included it in their online newsletter to gamers. In the call for participation in the study, gamers were asked to visit a password-protected website and complete the questionnaire.

Before filling out the questionnaire, all participants were informed about the general goals of the study (ie, to obtain an objective and real picture of online games and the online gamer community in contrast with the simplified and rather negative picture the media disseminates) and the time needed to complete it. They were assured about confidentiality and anonymity, and their informed consent was obtained by ticking a box if they agreed to continue and participate in the study. No personal information was collected or stored and no incentives were offered. Data collection took place between April and July 2009. The advertisements were displayed for approximately 3 months, whereas the newsletters including the call for participation were sent twice. According to the voluntary nature of participation, answering all the questions was not mandatory. The number of unique website visitors was not assessed at the time of the recruitment period, and the number of overlapping visitors between the 18 websites was also unknown; therefore, the view rate could not be calculated. In total, 7520 gamers visited the first page of the questionnaire and 4374 completed at least some of it. Consequently, the participation rate was 58.16%. Of these 4374 gamers, 3186 completed the entire survey including psychiatric symptoms, online gaming motives, and problematic online game use resulting in a completion rate of 72.84%. Consequently, all analyses were carried out on this latter subsample. Only completed questionnaires were analyzed. However, skipping answers was allowed; therefore, missing data were treated with full information maximum likelihood (FIML) method with MPlus 6.0.

### Measures

#### Sociodemographic Variables

Data relating to major sociodemographics were collected including age, gender, marital status, and education.

#### Gaming-Related Variables

Data were collected regarding weekly game time and preferred game type. Game type preference was obtained using the results of a latent profile analysis on the amount of time spent playing different game types described in a previous study that used the same sample (which was slightly bigger than the current sample because it also included those respondents who did not complete the Brief Symptom Inventory [BSI] placed at the end of the questionnaire) [43]. The majority of the sample (2517/3186, 79.00%) had a clear game type preference. Almost half of the sample (1466/3186, 46.01%) played almost exclusively with MMORPGs, whereas an additional 872 of 3186 gamers (27.37%) preferred MOFPSs. A small minority of the sample, 118 of 3186 gamers (3.70%), mostly played MMORTS games. The rest of the gamers (61/3186, 1.91%) played other online games (ie, sport games, puzzle games) or did not have a clear game type preference (669/3189, 21.00%). Therefore, the majority of the sample (2338/3186, 73.38%) could be categorized as either an MMORPG player or an MOFPS player. In the moderation analysis, only these 2 groups were focused on due to the low sample size of the remaining groups (ie, MMORTS players and gamers playing other online game types).

#### Psychiatric Symptoms

These were assessed using the Hungarian version of the BSI [45,46], a measure that assesses self-reported clinically relevant psychological symptoms. The 53-item questionnaire uses a 5-point Likert scale (from “not at all” to “extremely”). The BSI comprises 9 symptom dimensions: somatization, obsession compulsion, interpersonal sensitivity, depression, anxiety, hostility, phobic anxiety, paranoid ideation, and psychoticism. However, in the present study, the summarized measure—the Global Severity Index (GSI)—was applied to measure the intensity of general distress. Good reliability and validity has been found across various samples [45-47] as well as in this study (Cronbach alpha=.97).

#### Online Gaming Motives

These were assessed using the Motives for Online Gaming Questionnaire (MOGQ) [8]. This 27-item self-report measure assesses the full range of motives for online gaming. The 7 motivational dimensions assessed by MOGQ comprise social, escape, competition, skill development, coping, fantasy, and recreation. A brief description of each motivation has already been mentioned in the Introduction. In addition, a sample item for each motivation is as follows: social (eg, “...because I can meet many different people”), escape (eg, “...to forget about unpleasant things or offenses”), competition (eg, “...because it is good to feel that I am better than others”), skill development (eg, “...because it improves my skills”), coping (eg, “...because it helps me get rid of stress”), fantasy (eg, “...to be somebody else for a while”), and recreation (eg, “...because it is entertaining”). The MOGQ uses a 5-point Likert scale from “never” to “almost always/always” with higher scores indicating higher frequency of the respective motivational dimension. Internal consistencies were reported for all 7 dimensions, ranging from .79 to .90 [8].

#### Problematic Use of Online Games

This was assessed using the Problematic Online Gaming Questionnaire (POGQ) [29]. The POGQ is an 18-item self-report assessment tool with good psychometric properties based on both theoretical and empirical content. It assesses 6 dimensions of problematic gaming, namely preoccupation, overuse, immersion, social isolation, interpersonal conflicts, and withdrawal. The items are measured on a 5-point Likert scale (from “never” to “always”) and a summarized score can also be calculated that shows the severity of the problematic use (with higher scores indicating more serious problems being due to online gaming). A cut-off score of 66 points has been suggested to determine the proportion of gamers at high risk of problematic use. This cut-off score was determined empirically in the same article [29]. Conducting a latent profile analysis, 4 severity groups were identified and a cut-off score was calculated by using a sensitivity and specificity analysis based on the most severe group as the criterion group. The factor structure of the POGQ has been confirmed in further studies and high internal consistencies (.91) have also been reported [4,29].

### Statistical Analyses

Structural regression analyses within structural equation modeling (SEM) were used to test the proposed mediation models. Because the scales were not normally distributed, maximum likelihood estimation robust to nonnormality (MLR) was used in all SEM analyses [48]. Multigroup analyses were used to test gender and game type differences. To evaluate goodness of fit of the overall models, the chi-square goodness-of-fit statistic (with a *P* value <.05), the comparative fit index (CFI), the Tucker-Lewis fit index or nonnormed fit index (TLI or NNFI), root mean square error approximation (RMSEA) and its 90% confidence interval (90% CI), and the standardized root mean square residuals (SRMR) were used. For both CFI and TLI, values greater than 0.9 were considered a good fit, whereas both the values of RMSEA and SRMR should be less than 0.08 for an acceptable degree of fit [49,50]. Descriptive analyses were performed with the SPSS 20.0 statistical software package and all SEM analyses were performed with MPlus 6.0.

## Results

### Descriptive Statistics

The mean age of the sample (N=3186) was 21.1 years (SD 5.9 years, range 14-54 years), and the majority of the sample (2859/3186, 89.74%) were male. As previously mentioned, 1466 of 3186 gamers (46.01%) had a clear preference for MMORPGs and 872 of 3186 (27.37%) for MOFPSs. Among the MMORPG players, 240 gamers (16.37%) were female, whereas among MOFPS players only 20 gamers (2.29%) were female. Information regarding weekly game time and the proportion of gamers at high risk of problematic use is presented in Table 1.

It was assumed that the parameters of the proposed model might be moderated by players’ game type preference and gender. Therefore, descriptive statistics and group differences regarding the variables included in the model are presented in Tables 2 and 3.

Results demonstrated that MOFPS players reported significantly higher scores on competition, skill development, and coping motives, whereas MMORPG players scored significantly higher on fantasy, escape, POG, the GSI, and recreation.

**Table 1:** Weekly game time and proportion of gamers at high risk of problematic use for the overall sample, for males and females, and for multiplayer online first-person shooter (MOFPS) and massively multiplayer online role-playing games (MMORPGs) gamer types.

Gaming-related variables		Total sample, n (%) (N=3186)	Gender, n (%)		Game type preference, n (%)	
			Males (n=2859)	Females (n=327)	MOFPS players (n=872)	MMORPG players (n=1466)
**Weekly game time**						
	<7 hours	382 (12.00)	328 (11.48)	54 (16.5)	125 (14.4)	137 (9.36)
	7-14 hours	772 (24.25)	688 (24.09)	84 (25.7)	266 (30.5)	298 (20.36)
	15-28 hours	1102 (34.62)	1001 (35.05)	101 (30.9)	308 (35.4)	509 (34.77)
	29-42 hours	639 (20.08)	583 (20.41)	56 (17.1)	136 (15.6)	355 (24.25)
	>42 hours	288 (9.05)	256 (8.96)	32 (9.8)	36 (4.1)	165 (11.27)
Gamers at high risk of problematic use^a^		2.43	2.39	2.76	1.96	2.95

^a^ The proportion of gamers at high risk of problematic use was calculated using the established cut-off point (ie, 66) suggested in a previous article [29]. Note that only MOFPS and MMORPG gamers have been included because the MMORTS gamer group was very small and the remainder of the players could not be differentiated regarding their game type preference.

**Table 2:** Means, standard deviations (SD), and confidence intervals (CI) for multiplayer online first-person shooter (MOFPS) and massively multiplayer online role-playing games (MMORPGs) gamer types examined and for all players (MOFPS and MMORPG)^a^ and effect sizes (Cohen’s *d*).

Psychopathology- and gaming-related variables^b^		All (MOFPS & MMORPG) players (n=2338)		MOFPS players (n=872)			MMORPG players (n=1466)			Comparison of MOFPS and MMORPG players			
		Mean (SD)	95% CI	Mean (SD)	95% CI		Mean (SD)	95% CI		*t* (df)		*P*	*d*	
Global Severity Index		0.61 (0.61)	0.59-0.64	0.57 (0.57)	0.53-0.61		0.64 (0.64)	0.61-0.67		2.82 (1986.8)		.005	0.12	
POGQ Total score		36.13 (11.93)	35.65-36.62	34.99 (11.73)	34.21-35.77		36.82 (12.00)	36.20-37.44		3.59 (2326)		<.001	0.15	
**MOGQ**														
	Escape	1.93 (1.01)	1.89-1.97	1.83 (0.93)	1.76-1.88		2.00 (1.06)	1.95-2.05		4.09 (2019.5)		<.001	0.18	
	Coping	2.51 (1.08)	2.47-2.55	2.57 (1.12)	2.50-2.64		2.47 (1.05)	2.42-2.52		1.99 (1740.8)		.047	0.09	
	Fantasy	2.33 (1.13)	2.28-2.38	2.05 (1.00)	1.98-2.12		2.49 (1.17)	2.43-2.55		9.67 (2057.9)		<.001	0.40	
	Skill development	2.23 (1.14)	2.18-2.28	2.54 (1.21)	2.46-2.62		2.04 (1.05)	1.99-2.09		9.99 (1635.4)		<.001	0.44	
	Recreation	4.18 (0.87)	4.15-4.22	4.12 (0.90)	4.06-4.18		4.22 (0.85)	4.18-4.26		2.75 (1737.7)		.006	0.11	
	Competition	2.39 (1.19)	2.34-2.44	2.75 (1.22)	2.67-2.83		2.17 (1.11)	2.11-2.23		11.41 (1693.5)		<.001	0.50	
	Social	3.07 (1.20)	3.02-3.12	3.07 (1.21)	2.99-3.15		3.07 (1.19)	3.01-3.13		0.15 (2332)		.88	0.00	

^a^ Only MOFPS and MMORPG gamers have been included because the MMORTS gamer group was very small and the rest of the players could not be differentiated regarding their game type preference.

^b^ POGQ: Problematic Online Gaming Questionnaire; MOGQ: Motives for Online Gaming Questionnaire.

**Table 3:** Means, standard deviations (SD), and confidence intervals (CI) for both genders and for the total sample and effects sizes (Cohen’s *d*).

Psychopathology- and gaming-related variables^a^			Total sample (N=3186)		Males (n=2859)		Females (n=327)		Gender comparison		
			Mean (SD)	95% CI	Mean (SD)	95% CI	Mean (SD)	95% CI	*t* (df)	*P*	*d*
Global Severity Index			0.62 (0.62)	0.60-0.64	0.60 (0.61)	0.58-0.62	0.77 (0.69)	0.70-0.85	4.05 (387.4)	<.001	0.26
POGQ Total Score			35.89 (11.85)	35.48-36.30	35.86 (11.83)	35.43-36.30	36.12 (12.31)	35.78-37.46	0.37 (3171)	.71	0.02
**MOGQ**											
	Escape		1.91 (0.99)	1.88-1.94	1.87 (0.97)	1.84-1.90	2.28 (1.14)	2.16-2.40	6.22 (380.2)	<.001	0.39
	Coping		2.50 (1.07)	2.46-2.54	2.50 (1.08)	2.46-2.54	2.56 (0.99)	2.45-2.67	1.05 (3177)	.30	0.06
	Fantasy		2.33 (1.12)	2.29-2.37	2.28 (1.10)	2.24-2.32	2.77 (1.23)	2.64-2.90	6.80 (386.5)	<.001	0.42
	Skill development		2.25 (1.14)	2.21-2.29	2.26 (1.15)	2.22-2.30	2.17 (1.00)	2.06-2.28	1.54 (430.9)	.13	0.08
	Recreation		4.18 (0.88)	4.15-4.21	4.16 (0.88)	4.13-4.19	4.29 (0.82)	4.20-4.38	2.61 (416.5)	.009	0.15
	Competition		2.41 (1.18)	2.37-2.45	2.47 (1.18)	2.43-2.51	1.80 (0.93)	1.70-1.90	12.06 (456.4)	<.001	0.63
	Social		3.04 (1.20)	3.00-3.08	3.01 (1.20)	2.97-3.05	3.32 (1.18)	3.19-3.45	4.42 (3177)	<.001	0.26

^a^ POGQ: Problematic Online Gaming Questionnaire; MOGQ: Motives for Online Gaming Questionnaire.

Results also demonstrated that female gamers scored significantly higher on fantasy, escape, social, and recreation motives as well as on the GSI of psychiatric symptoms, whereas male gamers reported significantly higher scores only on competition motive. The zero-order correlations between the components of the mediation model along with internal consistencies (Cronbach alpha) for all scales and subscales are presented in Table 4.

All correlation coefficients were significant at least *P*<.001 according to Bonferroni correction except for GSI-MOGQ recreation (*P*=.61).

Because both the BSI and the POGQ are multidimensional scales, a comprehensive correlation matrix is also provided as a multimedia appendix containing all subscales of the BSI, the MOGQ, and the POGQ (see Multimedia Appendix 1).

**Table 4:** Zero-order correlations and Cronbach alphas (N=3186).

Psychopathology- and gaming-related variables	2	3	4	5	6	7	8	9	Cronbach α
1. Global Severity Index	.55	.51	.29	.36	.11	–.01	.20	.09	.97
2. POGQ Total Score		.51	.39	.40	.19	.15	.37	.26	.91
3. MOGQ Escape			.60	.61	.24	.18	.27	.29	.87
4. MOGQ Coping				.50	.41	.41	.38	.42	.84
5. MOGQ Fantasy					.30	.29	.28	.34	.83
6. MOGQ Skill development						.23	.38	.45	.89
7. MOGQ Recreation							.23	.35	.78
8. MOGQ Competition								.32	.90
9. MOGQ Social									.91

### Mediation Analysis

#### Overview

It was hypothesized that general psychiatric distress has both a direct and indirect effect (via the mediating effect of the 7 online gaming motives) on POG. Psychiatric distress was measured by the GSI and introduced in the model as a continuous observed variable. Problematic online gaming was measured by the summarized score of the POGQ and also introduced in the model as a continuous observed variable. Gaming motives were measured by the 7 factors of the MOGQ and introduced in the model as continuous latent variables. The proposed mediation models were tested with SEM. Because significant gender and gamer type differences were found in many of the variables included in the model, 3 different models were tested: (1) an overall model, (2) a separate multigroup analysis for the 2 gamer types, and (3) another multigroup analysis for males and females. The third model was carried out on the MMORPG gamer subsample because this group was the only one that had enough female players for comparison.

#### The Overall Model

**Figure 1:**
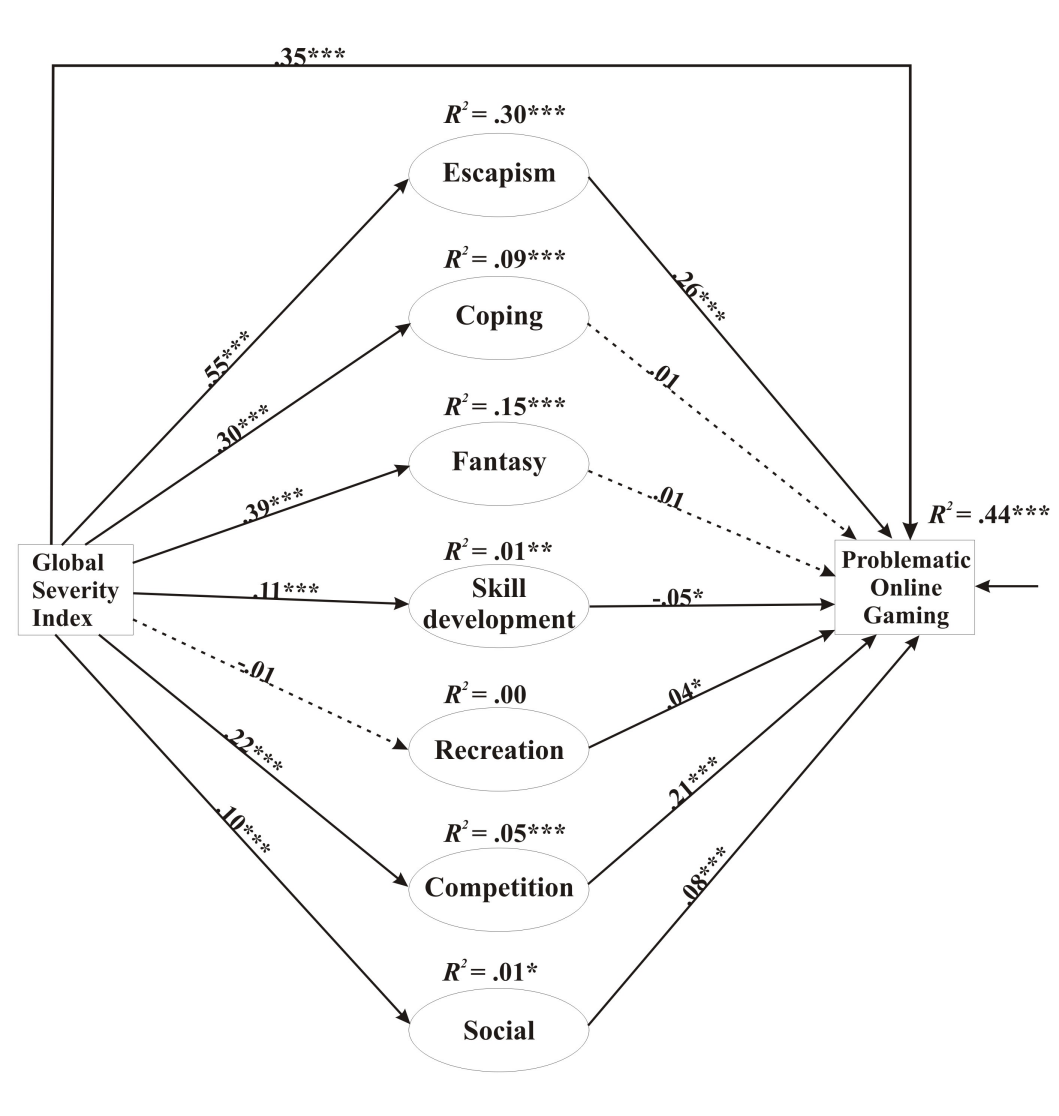
The overall mediation model with standardized path coefficients and the explained variance of the endogen variables (*R^2^*) (N=3186). All 7 mediator variables are latent variables. For clarity, indicator variables associated with them have not been depicted in this figure but were published in an earlier paper [8]. Also for clarity, the covariances between the errors of all mediator variables have not been depicted in the figure. Simple arrows: significant path coefficients, dotted arrows: nonsignificant path coefficients. *P<.05; **P<.01; ***P<.001.

The overall model had an adequate fit to the data (χ^2^_343_= 3217.8, *P*<.001; CFI=0.935; TLI=0.923; RMSEA=0.051, 90% CI 0.050-0.053; Cfit>0.90; SRMR=0.046). According to the results (see Figure 1), psychiatric symptoms had a significant direct effect on POG (standardized effect=.35, *P*<.001) as well as on all the gaming motives apart from recreation (standardized effects ranging from .10 to .55, *P*<.001). Psychiatric symptoms were significantly and strongly associated with escape, coping, and fantasy, and significantly but weakly associated with skill development, competition, and social motives. In relation to the association between gaming and problematic use, only escape and competition motives had a considerable effect size (standardized effect=.26 and .21, respectively), whereas social motives, skill development, and recreation motives had significant but low effect sizes. In relation to the indirect effect between psychiatric symptoms and problematic gaming, 4 paths were statistically significant: (1) psychiatric symptoms → escape → problematic gaming (standardized effect=.139, *P*<.001); (2) psychiatric symptoms → competition → problematic gaming (standardized effect=.046, *P*<.001); (3) psychiatric symptoms → social motives → problematic gaming (standardized effect=.008, *P*=.001); and (4) psychiatric symptoms → skill development → problematic gaming (standardized effect=-.006, *P*=.02). However, the latter 2 pathways had a negligible effect size. The mediation pathways added up a total standardized indirect effect size of .194 (*P*<.001). The proportion of the mediated effect in the total effect was 35%. Therefore, higher levels of psychiatric symptoms were associated with higher escape and competition motives that were associated with higher level of problematic use. All other indirect pathways were nonsignificant (*P*>.05) or had a negligible effect size (ie, <.01). The full model explained 44% of the total variance of POG.

#### Gamer Type Comparison

**Figure 2:**
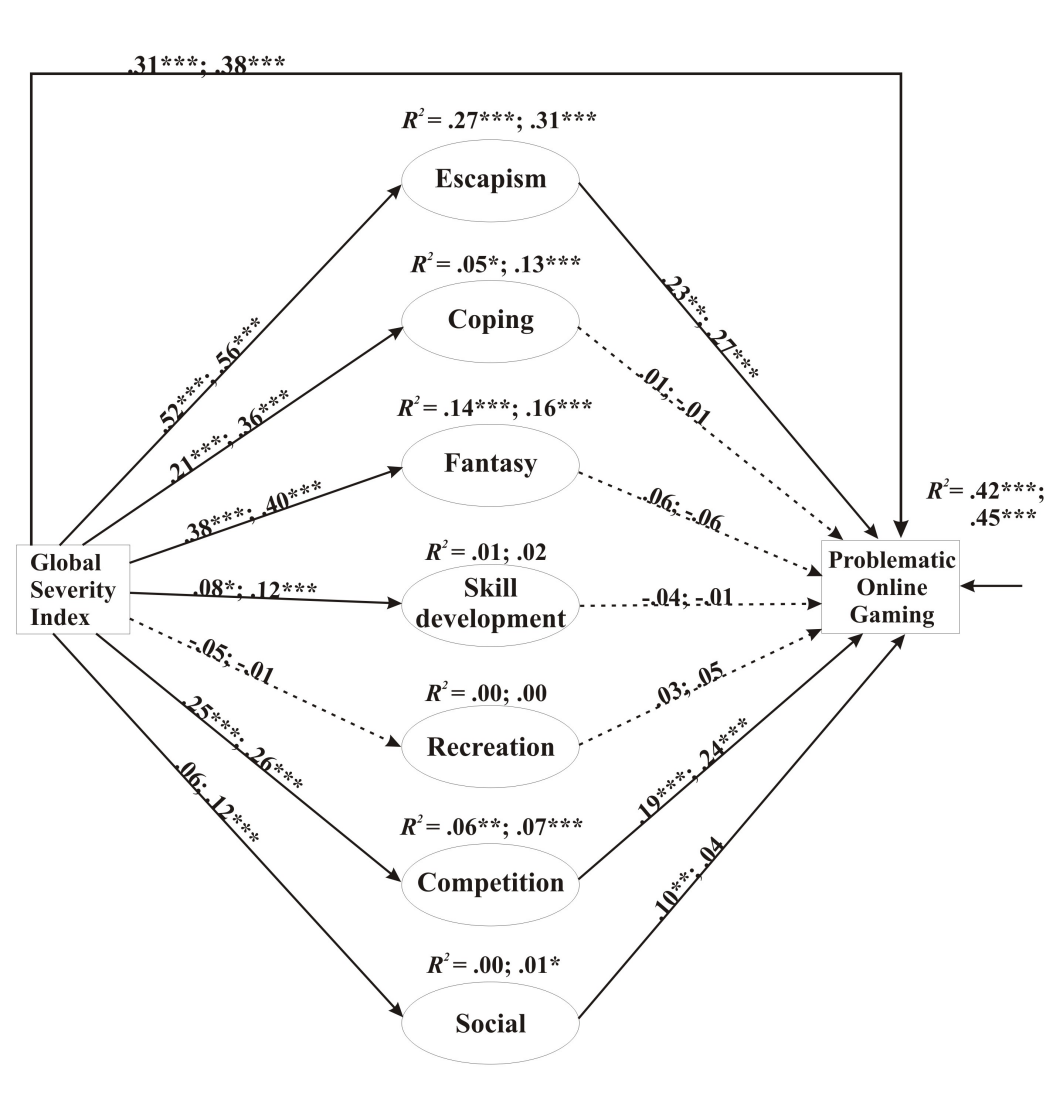
The mediation model and standardized path coefficients. Results of multigroup analysis and path coefficients across the 2 gamer types, multiplayer online first-person shooter (MOFPS) and massively multiplayer online role-playing games (MMORPGs), and the explained variance of the endogen variables (*R^2^*) (MOFPS: n=872; MMORPG: n=1466). The first (left) values describe MOFPS players, whereas the second (right) values describe MMORPG players. All 7 mediator variables are latent variables. For clarity, indicator variables associated with them have not been depicted but were published in an earlier paper [8]. Also for clarity, the covariances between the errors of all mediator variables have not been depicted in the figure. Simple arrows: significant path coefficients; dotted arrows: nonsignificant path coefficients. *P<.05; **P<.01; ***P<.001.

The model comparing MOFPS and MMORPG players also had an adequate fit to the data (χ^2^_726_=3120.7; MOFPS: χ^2^_726_=1343.6; MMORPG: χ^2^_726_=1777.0, *P*<.001; CFI=0.926; TLI=0.918; RMSEA=0.053, 90% CI 0.051-0.055; Cfit>0.90; SRMR=0.051). Overall, the results (see Figure 2) were fairly similar to the first model (Figure 1). The psychiatric symptoms had a significant direct effect on POG in both groups and the psychiatric symptoms → escape → POG and the psychiatric symptoms → competition → POG indirect pathways were significant and had a considerable effect size in both groups. In the case of MOFPS players, the standardized effect size of the direct pathway was .314 (*P*<.001), whereas the standardized effect size of the mediation pathway was .194 (*P*<.001) that amounted to 38.2% of the total effect size. The mediation pathway via escape had a standardized effect size of .119 (*P=*.001) and the one via competition had an effect size of .048 (*P*<.001). Neither of the other indirect pathways were significant. The full model explained 42% of the total variance of POG. In the case of MMORPG players, the standardized effect size of the direct pathway was .376 (*P*<.001), whereas the standardized effect size of the mediation pathway was .191 (*P*<.001) that amounted to 33.7% of the total effect size. The mediation pathway via escape had a standardized effect size of .152 (*P*<.001) and the one via competition had an effect size of .063 (*P*<.001). The full model explained 45% of the total variance of POG. Neither of the other indirect pathways was significant. The comparison of the 2 player types according to the Wald test showed no significant differences in the model.

#### Gender Comparison Between Massively Multiplayer Online Role-Playing Game Players

**Figure 3:**
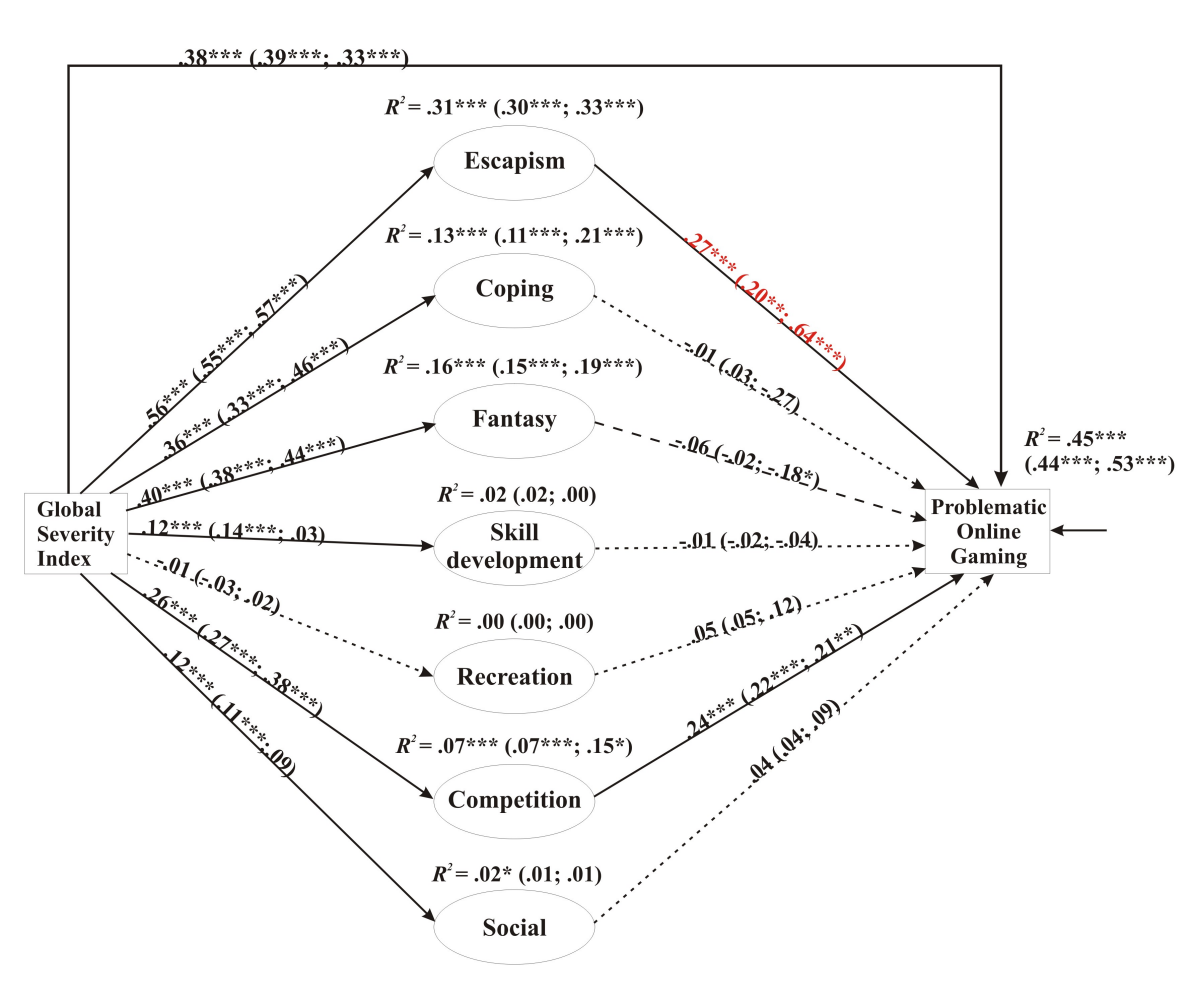
The mediation model and standardized path coefficients. Results of multigroup analysis and path coefficients across both genders (males/females) and the explained variance of the endogen variables (*R^2^*) (males: n=1226; females: n=240). The first values (left of the brackets) describe all MMORPG players. The first (left) values in the brackets describe male MMORPG players, whereas the second (right) values describe female MMORPG players. The color red indicates a significant difference between male and female players according to the Wald test. All 7 mediator variables are latent variables. For clarity, indicator variables associated with them have not been depicted in this figure but were published in an earlier paper [8]. Also for clarity, the covariances between the errors of all mediator variables have not been depicted in the figure. Simple arrows: significant path coefficients; dotted arrows: nonsignificant path coefficients; dashed arrow: nonsignificant path coefficients for males, significant path coefficients for females. *P<.05; **P<.01; ***P<.001.

Given that the proportion and sample size of female players were only considerable among MMORPG players, the third model was carried out only on this group (Figure 3). The model fitted the data properly (χ^2^_726_=2232.0; males: χ^2^_726_=1531.3; females: χ^2^_726_=700.7; *P*<.001; CFI=0.929; TLI=0.920; RMSEA=0.053, 90% CI 0.051-0.056; Cfit>0.90; SRMR=0.049). In relation to MMORPG players (Figure 3), the results were also similar to the results of the overall model (Figure 1). Psychiatric symptoms again had a direct effect on POG with a standardized effect size of .38 (*P*<.001). Again, only the mediation pathways via escape and competition were significant with standardized effect sizes of .153 (*P*<.001) and .063 (*P*<.001), respectively. The standardized effect size of the indirect link between psychiatric symptoms and POG was .191 (*P*<.001) that amounted to 33.7% of the total effect size. The full model explained 45% of the total variance of POG.

Results relating to gender differences showed a significant difference in the escape → POG direct link between male and female MMORPG players. The standardized direct effect size of this link was .20 (*P=*.001) for men and .64 (*P*<.001) for women. The group difference between males and females was significant (Wald test=6.11, *P*=.01). As a result, the psychiatric symptoms → escape → POG mediator pathway for female players had a much higher standardized effect size (standardized effect=.368, *P*<.001) than the one for male players (standardized effect=.111, *P*=.001). This also led to a stronger indirect link between psychiatric symptoms and problematic gaming for female players (standardized effect=.253, *P*<.001) than for male players (standardized effect=.175, *P*<.001). The total explained variance of POG by the model was also slightly higher for female players (53%) than for males (44%).

## Discussion

### Principal Results and Comparison With Prior Work

The results of the present study suggest that psychiatric symptoms are both directly and indirectly (via escape and competition motives) negatively associated with POG. The mediator effect of gaming motives amounts to approximately 35% of the total effect. To the authors’ knowledge, this is the first study to statistically unravel the complex association between psychiatric symptoms, online gaming motivations, and problematic use using a SEM framework. The results relating to the associations between motives and problematic use are similar to previous findings in the psychological gaming literature. For instance, Yee [11] and Billieux and colleagues [9] found that escapism was the best predictor of problematic use in the case of MMORPG players followed by advancement motivation (ie, the desire to progress rapidly in the game—“level up”—and become powerful).

Although advancement and competition motives are not the same, they are related to each other via a common connection to achievement and performance. Progressing rapidly and gaining power eventually become a way to be competitive, a way to complete goals successfully, and a way to defeat others. However, the advancement motive was developed in studies focusing on MMORPGs where “leveling up” is of a particular importance due to the persistence of the virtual world. On the other hand, the competition motive used in the present study is related to online games in general including games such as multiplayer online first-person shooter games or strategy games, in addition to MMORPGs. Other studies [12,13] have also reported a strong association between escapism and problematic gaming as well as achievement and problematic use. Achievement is the higher order factor in Yee’s [11] motivational model comprising 3 subdimensions (ie, advancement, mechanics, and competition), whereas in the study by Zanetta Dauriat [13], the motive with the same name refers to the need for being competitive, to obtain fame and recognition, and to be member of a top guild. Furthermore, our results relating to the association between psychiatric symptoms and motives are also in-line with previous findings. For instance, Hagström and Kaldo [51] reported that among all online gaming motives, escapism showed the strongest relationship with psychological distress.

In addition to confirming previous findings in the gaming literature, as predicted the present study showed that the same 2 motives (escape and competition) mediated between psychiatric symptoms and problematic gaming. Playing games to escape everyday difficulties appears to be a motivating behavior that can ease psychiatric distress, and thus extends self-medication theory [52] to online gaming. This theory states that substance use is a coping strategy through which users try to compensate their psychiatric distress and attain emotional stability. This compensatory behavior then contributes to the development and maintenance of the problem behavior. The findings outlined in the present study also strengthen the inclusion of escapism as an individual criterion for Internet Gaming Disorder in *DSM-5* [6].

The second mediator variable in the present study was competition. Despite the fact that competition is usually considered a healthy and adaptive behavior, our findings suggest that in some cases it can also be a pathological factor. This has also been reported in the literature on problem gambling in which problem gamblers have been shown to be more competitive than nonproblem gamblers [53]. Gamers whose psychiatric distress level is high might use online gaming as a source for achievement through defeating other players and winning in general. If games are the only sources that maintain and boost their self-confidence and self-efficacy, and thus become a replacement for real life competition and achievement, the activity appears to increase the risk of developing a problematic behavior. However, this reasoning needs further confirmation.

In contrast to prestudy expectations, coping did not mediate between psychiatric distress and problematic gaming. Earlier motivational research yielded the surprising finding that although highly correlated (.60), coping and escape are distinct motives [8], and the present study strengthened the argument that these 2 motives have different mechanisms of action. The results of the present study suggest that in contrast to playing to escape everyday problems, gaming can also be used as an adaptive coping strategy for stress release or tension reduction without necessarily leading to problematic use. A possible explanation might be that different underlying mechanisms lie behind the 2 strategies. Avoiding real life problems (ie, escape) only alleviates the perceived stress for a short time, retaining or further multiplying the original problem (ie, stress source). On the other hand, channeling everyday stress, tension, or aggression into gaming (ie, coping) can be an active coping mechanism where at least some extent of the perceived stress is dissipated while playing. However, this is speculative and requires further research.

The recreational use of online games was related neither to psychiatric symptoms nor to problematic use of games. This suggests that playing online games can be a healthy form of entertainment if it is used moderately and balanced with other leisure time activities (ie, sports). This result also serves as counterweight for media scaremongering that often exaggerates the potential dangers of video games [54].

This model in the present study was found to be invariant across game type preference (ie, MOFPS or MMORPG), but varied significantly between males and females in the case of MMORPG players. As expected, females were characterized by a stronger link between escape and POG, and also had higher escape scores than males. This latter result is in-line with Yee’s findings [55] that examined the motivational background of MMORPG players and also found that female players scored higher on the escape motive than male players. However, the present study suggests that this higher inclination for escape motivation among females is linked to a higher risk of problematic use. In contrast to the other prestudy assumptions, no gender differences were found regarding competition and social motives.

In addition, it is important to point out that the proportion of female players in the present sample is much lower than the proportion of women that play video games in the general population (ie, approximately 40% [4,18]). This is most likely due to the online data collection method in which participation is voluntary. In online gamer samples, the proportion of female players is usually quite low (approximately 10%-20% [55,56]). The reason might be that the so-called “hard-core” gamers are more interested in participating in such research studies and that the proportion of hard-core female players is lower than the proportion of hard-core male players. Therefore, it is important to acknowledge that these findings apply more to those female gamers who play seriously (in a hard-core or “masculine” gamer manner) than to the average female (casual) gamers in the general population [57,58].

### Limitations

Despite the advantage of a large sample size, the self-selected and self-reported nature of the Hungarian-only data need to be taken into consideration when generalizing the results (especially because recent research has shown that self-selection of MMORPG players affects the sample’s representativeness [59]). Consequently, there is a clear need for future observational and clinical studies to confirm the findings of the present study in other nationalities of gamers. The cross-sectional study design should be also borne in mind when applying the findings because causation and directionality of findings cannot be confirmed. Consequently, future studies should also use longitudinal or experimental design to establish causal relations regarding the proposed model. Furthermore, it is theoretically feasible to construct alternative models that act in the opposite direction (ie, POG leading to psychiatric distress) and/or that both models may not be mutually exclusive (that for some people psychiatric distress leads to POG and vice versa for others). Such possibilities should also be empirically tested in future studies.

### Implications for Prevention and Treatment

The present study has some direct implications for prevention and treatment. There is little reason for parents, educators, and health professionals to be concerned or worried about the recreational use of online games. Neither should they necessarily be concerned about playing as a way to cope with day-to-day stress or tension. However, playing excessively as a way to avoid real-life problems or to defeat other players should receive attention because such motivations may lead to negative (addiction-like) real-life consequences. Therefore, exploring gaming motivations both on the individual and group level are likely to be helpful in the preparation of prevention and treatment programs concerning problematic gaming.

## Multimedia Appendix 1

Correlation matrix including the nine Brief Symptom Inventory (BSI) subscales, the seven Motives for Online Gaming Questionnaire (MOGQ) subscales and the six Problematic Online Gaming Questionnaire (POGQ) subscales.

## Abbreviations

BSI: Brief Symptom Inventory
CFI: comparative fit index
FIML: full information maximum likelihood
GSI: Global Severity Index
MMORPG: massively multiplayer online role-playing games
MOFPS: multiplayer online first-person shooter
MMORTS: massively multiplayer online real-time strategy
MOGQ: Motives for Online Gaming Questionnaire
NNFI: nonnormed fit index
POG: problematic online gaming
POGQ: Problematic Online Gaming Questionnaire
RMSEA: root mean square error approximation
SEM: structural equation modeling
SRMR: standardized root mean square residuals
TLI: Tucker-Lewis fit index
